# Real-Time Online Goal Recognition in Continuous Domains via Deep Reinforcement Learning

**DOI:** 10.3390/e25101415

**Published:** 2023-10-04

**Authors:** Zihao Fang, Dejun Chen, Yunxiu Zeng, Tao Wang, Kai Xu

**Affiliations:** College of Systems Engineering, National University of Defense Technology, Changsha 410000, China; fangzihao21@nudt.edu.cn (Z.F.); chendejun18@nudt.edu.cn (D.C.); zengyunxiu@nudt.edu.cn (Y.Z.); wangtao1976@nudt.edu.cn (T.W.)

**Keywords:** online goal recognition, deep reinforcement learning, continuous domain, communication constraints, information entropy

## Abstract

The problem of goal recognition involves inferring the high-level task goals of an agent based on observations of its behavior in an environment. Current methods for achieving this task rely on offline comparison inference of observed behavior in discrete environments, which presents several challenges. First, accurately modeling the behavior of the observed agent requires significant computational resources. Second, continuous simulation environments cannot be accurately recognized using existing methods. Finally, real-time computing power is required to infer the likelihood of each potential goal. In this paper, we propose an advanced and efficient real-time online goal recognition algorithm based on deep reinforcement learning in continuous domains. By leveraging the offline modeling of the observed agent’s behavior with deep reinforcement learning, our algorithm achieves real-time goal recognition. We evaluate the algorithm’s online goal recognition accuracy and stability in continuous simulation environments under communication constraints.

## 1. Introduction

Goal recognition is a form of intent recognition that differs from plan recognition in that it focuses on determining the high-level objective pursued by an agent, rather than its specific action plan. Its aim is to infer the top-level goal of an agent based on input observation sequences and output an explanation of the observation sequence [1]. Currently, goal recognition has been applied in various fields, including intelligent assistants [2,3], autonomous driving [4,5], robot navigation [6,7], military confrontation [8], and more [9]. For example, in the application of goal recognition in a smart home assistant [2,3], the input observation sequence consists of a series of actions performed by a person as observed by a camera. By using a goal recognition algorithm, the system can, for example, identify the goal of an elderly person as needing to go to the kitchen. It effectively assists systems in understanding and predicting the intentions and behavior of agents, as well as assisting humans in completing complex tasks. As such, goal recognition has become an increasingly important area of research with practical applications in a variety of domains.

The task of goal recognition can be addressed through plan-based and learning-based intent recognition methods. Plan-based methods involve decomposing the goal recognition problem into planning and recognition components [10]. The recognition component selects the most probable goal explanation for each observed action by utilizing the planner. However, this approach is limited by challenges such as the need for a well-defined domain description, noisy observations, and high online computational complexity. Therefore, plan-based methods have certain drawbacks that need to be addressed. In contrast to plan-based methods, recent research has proposed a reinforcement learning-based framework for goal recognition [11] that adopts an evaluative approach. Unlike traditional plan-based methods that compare the generated optimal plan with the observation sequence, the reinforcement learning-based approach learns the corresponding action Q-table for each state through Q-learning. This approach effectively addresses the drawbacks of plan-based recognition methods and outputs the most likely goal by evaluating the observation sequence. However, the proposed method has only been tested in discrete Planning Domain Definition Language (PDDL) [11,12] and is not suitable for continuous environments such as robot navigation, where the Q-table cannot fully describe the infinite and continuous action space. Furthermore, the method has only been evaluated through offline state recognition testing [13,14], and the challenges of online recognition using incremental inputs of observation sequences and communication constraints have not yet been addressed.

This paper introduces a novel framework called Goal Recognition as Deep Reinforcement Learning (GR_DRL), which employs deep reinforcement learning for achieving online goal recognition tasks in the context of robot navigation in a continuous domain. The framework utilizes a deep neural network to output the reward values of actions in a continuous action space. By utilizing the cumulative Q-measure based on the GR_RL framework [11], the proposed approach enables online goal recognition in a continuous domain.

This paper presents three significant contributions. First, we extend the reinforcement learning-based goal recognition framework GR_RL proposed in [11] to the continuous domain. Our framework consists of two main stages: modeling opponent behavior using the TD3 deep reinforcement learning algorithm and learning the behavior policy of the agent in continuous environments, with the navigation task serving as an example. During the goal recognition process, a deep neural network in the trained model is utilized to evaluate continuous observation–action sequences and perform online inference to determine possible goals. Second, we address various issues encountered during online recognition in real robot navigation environments, such as sensor failures, asynchronous sampling rates, and communication interference (observation noise) [13]. Third, we evaluate the online goal recognition speed of the GR_DRL algorithm in continuous navigation environments. In summary, our paper proposes an online goal recognition algorithm based on deep reinforcement learning and validates it in robot navigation tasks in a continuous domain. Our experiments demonstrate that the GR_DRL algorithm achieves excellent recognition accuracy and robustness while maintaining fast online recognition speed.

This paper is structured as follows: We begin by introducing the relevant concepts and methods of goal recognition and formally defining the online goal recognition problem in a continuous domain. We then present the framework and model design for solving goal recognition problems based on deep reinforcement learning. Subsequently, we describe the experimental environment for online robot navigation tasks in the continuous domain, outline the experimental assumptions, and provide a qualitative and quantitative analysis of the experimental results. Finally, we analyze and discuss the advantages and limitations of the experimental results and the model and identify future research directions.

## 2. Background

### 2.1. Goal Recognition Problem

Goal recognition is a crucial aspect of intention recognition, which can be classified into action, plan, and goal recognition based on the recognized level of abstraction [15]. Action recognition operates at the lowest level, taking noisy sensor signals as input and identifying the lowest-level actions, such as turning left or going straight. Goal recognition, on the other hand, operates at the highest level, taking a sequence of discrete symbols as input and identifying the top-level goal that can explain the observed sequence, such as a specific destination in a navigation task. Plan recognition serves as a bridge between action and goal recognition, also taking a sequence of discrete symbols as input. It identifies both the top-level goal that explains the observed sequence and each action that leads to the completion of the top-level goal, such as a specific destination and the ordered sequence of agent movements in a navigation task [16]. Since the top-level goal in plan recognition is usually the final goal of the task, many methods in plan recognition are also applicable to goal recognition. Therefore, our paper will introduce them together.

Initially, we identify the common consensus within the field to define the GR problem based on intention recognition (Meneguzzi and Pereira 2021; Mirsky, Keren, and Geib 2021) [15,17]. As formally defined in Definition 1, the GR problem consists of a tuple, where D represents the domain theory, G represents the set of potential goals, and O represents a sequence of observations. The objective of the GR problem is to find the best goal g that explains the observation sequence.

**Definition** **1.** ***(Goal recognition problem)** A goal recognition problem is a tuple T=〈D,G,O〉 composed of a domain theory D, a set of potential goals G, and a sequence of observations O. The objective of the problem is to identify a goal g∈G that provides an explanation for the given observation sequence O.*

The current dissimilarities among different approaches primarily stem from the formulation of the domain theory and the techniques employed for interpreting the observation sequence O. For example, planning-based methods utilize domain knowledge derived from planning to establish the domain and convert the recognition process into a planning procedure. The most probable goal that can explicate the observation sequence is deduced by comparing the observation sequence and the planning sequence [10,18]. Conversely, learning-based methods primarily utilize historical or interactive data to acquire knowledge about the domain of the goal individual [19,20].

### 2.2. Goal Recognition as Planning

The concept of planning-based goal recognition was first presented by Ramírez and Geffner in 2009, through their work on Plan Recognition as Planning (PRAP) [10]. Subsequent advancements in this field have consistently adopted the same fundamental principle, which involves utilizing classical planning concepts to calculate a probability distribution over a set of potential plans or goals [6,10]. The recognition process is modeled as the reverse of the planning process, which involves decomposing the traditional recognition process into two distinct components: recognition and planning. The planner is responsible for generating feasible paths, while the recognizer processes the observation sequence, invokes the planner, and computes the probability distribution.

**Definition** **2.** ***(Goal Recognition as Planning)** A goal recognition as planning problem is defined by a tuple T=〈Dp,G,O〉. Here, the planning-based domain theory Dp is a tuple $Dp=\langle F,s_{0},A\rangle$, where $s_{0}\subseteq F$ denotes the initial state and A is a set of actions. Each action a∈A has preconditions Pre(a)⊆F and lists of fluents Add(a)⊆F and Del(a)⊆F that describe the effects of the action a in terms of fluents that are added and deleted from the current state. Additionally, actions have non-negative costs c(a), and the cost of a plan is defined as $c\left(\pi \right)=\sum_{i}^{}c\left(ai\right)$. Meanwhile, G denotes a set of possible goals, where each goal g⊆F, and O represents a sequence of observations $O=O_{1},\cdots O_{m}$, where each observation $O_{i}\in A$ is a sequence of actions that have been observed.*

Currently, planning-based methods for goal recognition are based on the idea of using Bayesian inference to calculate the posterior probability of a goal [10,21], assuming that the prior probabilities of each possible goal in the goal set are given in the problem:

(1) P(G∣O)=αP(O∣G)P(G)

Therefore, the focus of the goal recognition problem is transformed into an estimation problem for probabilities, and planning-based methods utilize planning techniques to address this issue.

Ramírez and Geffner (2010) [10] postulate that agents act with complete rationality, utilizing strictly optimal plans that minimize costs to achieve their objectives. Furthermore, they assume that the probability of a goal becoming the actual objective of an agent can be estimated by the cost difference between the optimal plan that includes a given observation sequence O and the optimal plan that does not include O, while achieving a given goal g⊆G. This is because the optimal plan that does not need to satisfy the requirement of including O, according to the planning domain, is a fully rational plan from the given initial state to the given goal *g*. Therefore, when the cost of the optimal plan that includes O is higher, it implies that the agent is further away from the true objective. The authors present a method to compute P(O∣g) as follows:

(2) $$\Delta \left(g\right)=c\left(O,g\right)-c\left(\bar{O},g\right)$$

(3) $$P\left(O|g\right)=\alpha \frac{\exp\{-\beta \Delta (g)\}}{1+\exp\{-\beta \Delta (g)\}}$$

where α is the normalization factor and $\Delta \left(g\right)=c\left(O,g\right)-c\left(\bar{O},g\right)$ represents the cost difference between the optimal plan that satisfies observation O for goal *g* and the optimal plan that does not satisfy O for goal *g*. The costs c(O,g) and $c(\bar{O},g)$ can be calculated using classical planning systems.

Many studies have utilized automated planning techniques to conduct research on goal recognition, building on the foundational computational principles outlined above [10,18,22]. In the context of the domain hypothesis, Ramírez and Geffner (2011) and Oh et al. (2011) [23] investigated the stochastic variability of planning domains Dp, utilizing Markov models to represent this variability. Competitive relationships in goal recognition can be broadly categorized into three types, namely keyhole, intended, and adversarial recognition [24]. Intended recognition refers to the recognition process, where the observed agent is conscious of the recognition process and is often cooperative with the recognition process. Adversarial recognition, on the other hand, refers to instances where the observed agent is aware of the recognition process but chooses not to cooperate with it. Keyhole recognition describes a recognition process where the observed intelligent agent is unaware of the recognition process, and where the recognition process faces the challenge of partially observable inputs. While most goal recognition methods based on planning [7,10,25] are based on discrete domains, Vered and Kaminka [13,14,26] provided the first formal definition of goal recognition in continuous domains, which has since been further explored in subsequent studies [8].

**Figure 1:**
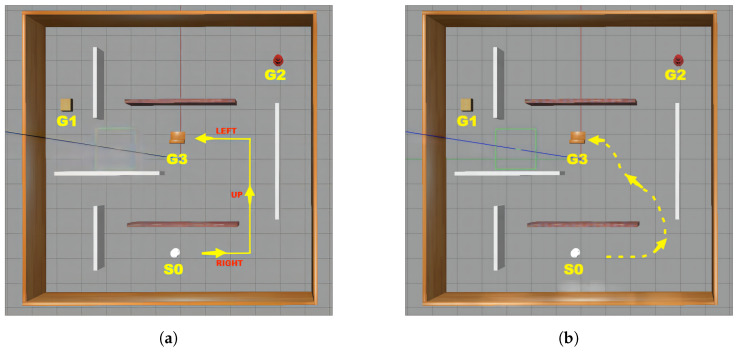
Continuous and discrete domains for the navigation environment: (**a**) discrete domain; (**b**) continuous domain.

**Definition** **3.** ***(Continuous and Discrete Domains)** In a continuous domain model D, the state space S is defined as a subset of an n-dimensional Euclidean space, denoted as $S\subset R^{n}\left(n\geq 2\right)$. This type of domain model is generally used to represent environments with two or three dimensions. The action space A of a continuous domain is a discrete set of actions, which can be infinite, and encodes a transition function between states. In contrast, both the states and actions in discrete domains are discrete. For example, for the navigation environment illustrated in Figure 1, the discrete domain can be represented by an action space A=〈left,right,up,down〉, an initial state $s_{0}$ = *〈*(robot loc-0-0), (box loc-4-5)…*〉*, and a sequence of observations O, represented by a yellow dashed arrow. In contrast, the continuous domain’s action space can be represented by continuous values of x, y, z, and θ. For the same navigation environment, the initial state $s_{0}$ can be represented as $\langle x\left(r\right)=0,y\left(r\right)=0.5,z\left(r\right)=0.1,\theta \left(r\right)=45^{\circ }\rangle$.*

The majority of planning-based approaches [7,10] have implemented an offline input method, which results in low efficiency when dealing with online incremental observation sequence inputs. This necessitates the repeated invocation of the offline goal recognition algorithm. Vered and Kaminka were the first to propose an efficient online goal recognition method in their work [13,14]. The main difference between the online and offline goal recognition methods lies in the input approach for the observation sequence O: the offline input approach involves providing the entire observation sequence to the recognition algorithm before execution, whereas the online goal recognition method involves providing the observation sequence O incrementally multiple times to the goal recognition algorithm.

### 2.3. Goal Recognition as Learning

Artificial intelligence and machine learning have advanced rapidly, leading to the emergence of a new paradigm for goal recognition based on learning theory. This paradigm can be categorized into two broad groups: model-based and model-free approaches, based on the learning method employed [27,28].

Model-based goal recognition focuses on learning the action model and domain theory of the recognizer. Amir et al. [20,29,30,31] employed various learning methods to study behavior models, but have not established a link between these models and the recognizer’s strategy. Zeng et al. [32] used inverse reinforcement learning to learn the recognizer’s reward and implemented a Markov-based goal recognition algorithm. However, for goal recognition, it is not necessary to learn the reward for the transition between all actions. To extract useful information from the image-based domain and perform goal recognition, Amado et al. [20] used a pre-trained encoder and LSTM network to represent and analyze observed state sequences, rather than relying on actions. Additionally, by training an LSTM-based system to recognize missing observations about states, Amado et al. [19] achieved improved performance for the model-based goal recognition method based on learning.

In contrast, model-free goal recognition based on learning requires no model and only utilizes the observed action sequence and initial state as input. Borrajo et al. [27,33] studied goal recognition using XGBoost and LSTM neural networks, which only employ observation sequences without any domain knowledge. However, they trained specific machine learning models for each goal recognition instance and used specific instance datasets for training and testing. M. Chiari [34] proposed a recognition network GRNet for goal recognition using an improved RNN network, which only requires the input of observed action data. The RNN network outputs the probability of the goal in the planning domain and has achieved good results in discrete PDDL planning examples.

Finally, Amado et al. [11] proposed a model for goal recognition behavior modeling based on reinforcement learning Q-learning, which combines model-free reinforcement learning with the latest goal recognition algorithms.

**Definition** **4.** ***(Goal Recognition as Reinforcement Learning)** A goal recognition as reinforcement learning problem is defined by a tuple $T=\langle D_{l},G,O\rangle$, where the domain theory $D_{l}$ is divided into two types: utility-based $T_{Q}\left(G\right)$ or policy-based $T_{\pi }\left(G\right)$. A utility-based domain theory $T_{Q}\left(G\right)$ is represented by a tuple (S,A,Q), where Q is a set of Q-functions ${\left\{Q_{g}\right\}}_{g\in G}$. On the other hand, a policy-based domain theory $T_{\pi }\left(G\right)$ is represented by a tuple (S,A,π), where π is a set of policies ${\left\{\pi_{g}\right\}}_{g\in G}$.*

They converted the planning task described in the traditional planning language PDDL into a reinforcement learning environment, allowing for the direct learning of the recognizer’s utility function or policy. They also proposed utility-based $T_{Q}\left(G\right)$ and policy-based $T_{\pi }\left(G\right)$ domain theories and used the metric of accumulated Q-value for reasoning. And in the context of goal recognition, the use of policy-based domain theory $T_{\pi }\left(G\right)$ can be replaced by a utility-based domain theory $T_{Q}\left(G\right)$. This replacement can be achieved by generating a softmax policy $\pi_{g}$ based on $Q_{g}$ for each goal *g*, as shown in Equation (4).

(4) $$\pi_{g}\left(a|s\right)=\frac{Q_{g}\left(s,a\right)}{\sum_{a^{\prime }\in A}Q_{g}\left(s,a^{\prime }\right)}$$

**Figure 2:**
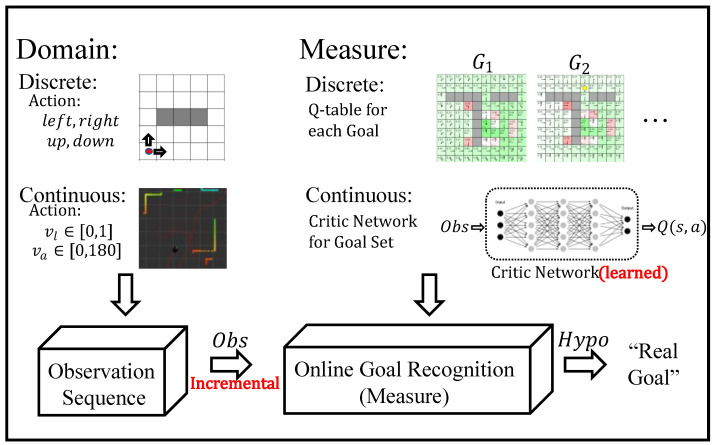
This figure illustrates the online goal recognition framework based on reinforcement learning in continuous domains. The red text highlights the key characteristics of the goal recognition algorithm in online continuous domains, particularly in terms of the comparison between discrete and continuous domains regarding action space and evaluation methods.

This approach effectively leverages reinforcement learning techniques to acquire domain models and mitigate the impact of noise on goal recognition by employing an evaluative strategy, thereby enhancing the speed of goal identification. However, the existing framework is constrained to finite and discrete action spaces, rendering it inadequate for addressing the challenges posed by infinite state and action spaces in continuous domains. To overcome this limitation, this study introduces a novel goal recognition framework based on deep reinforcement learning tailored for online continuous environments. The proposed framework investigates the learning process in continuous domains characterized by infinite action and state spaces using deep reinforcement learning. Furthermore, it explores methods for providing the most credible goal explanations for observed sequences. The accompanying Figure 2 elucidates the fundamental disparities between continuous and discrete domains within the context of the reinforcement learning framework.

## 3. The Goal Recognition as Deep Reinforcement Learning Framework

Our framework primarily comprises two integral modules. Firstly, an interactive learning methodology is employed for the evaluation of offline policy networks. Secondly, there is the incremental incorporation of observation sequences, concurrent with online goal inference and recognition. The workflow of this framework is visually depicted in Figure 2.

**Figure 3:**
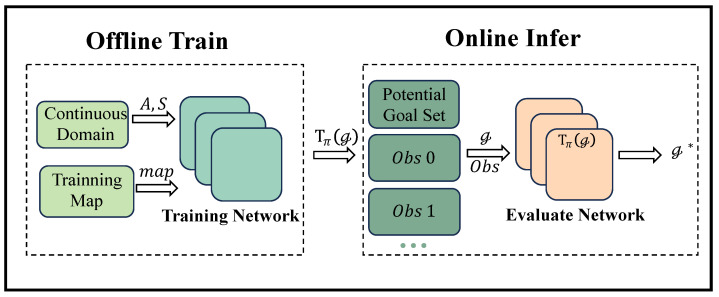
The proposed framework for GR as DRL.

Figure 3 illustrates the algorithmic flow framework for offline training and online inference proposed for GR as DRL.

The initial module of the framework takes a training map and the continuous action space denoted as A and the state space S corresponding to the observed agent as inputs. This component generates a domain-theoretic representation $T_{\pi }\left(G\right)$, which undergoes refinement through training within the policy evaluation network.

The second module encompasses an online incremental goal recognition procedure that progresses with each discrete time step. It continuously receives the latest observation values, represented as $\langle s,a\rangle$, derived from the observation sequence. Subsequently, a potential goal set G and the updated observation data, denoted as $O=\langle s_{0},a_{0},s_{1},a_{1},\ldots \rangle$, are fed into the online goal recognition module. This module then determines the most probable goal, denoted as $g^{*}$, which provides the most coherent explanation for the observation sequence O.

In the online inference model, we employ a distance evaluation method, akin to planning-based approaches, as demonstrated in Equation (5).

(5) $$g^{*}=\underset{g\in G}{\arg\min}\mathrm{Distance}\left(Q_{\pi_{g}},O\right)$$

The policy evaluation network $Q_{\pi_{g}}$, trained offline through reinforcement learning, generates Q-values for all possible goal *g* in the goal set G based on observation sequences O. According to the assumption of a rational agent, the goal $g^{*}$ with the highest Q-value is considered the most likely.

**Module One: Offline Training.** We initiate the offline training phase by training the behavioral policy of the intelligent agent within a continuous domain. This training process involves planning navigation tasks for randomly selected goal *g* within the training map, with the objective of developing a rational navigation agent proficient in continuous domains. As outlined in Algorithm 1, this procedure results in the creation of a domain-theoretic representation $T_{\pi }\left(G\right)$ based on the network architecture.

To commence the training, it is essential to initialize and optimize various training parameters, including reward functions r(s,a) and iteration counts *T* (line 1–3), similar to other deep reinforcement learning algorithms. During each training iteration, we randomly generate goal points within the map and compute reward values for states S and actions A based on the defined reward function. Subsequently, we update the policy network to generate the domain-theoretic representation $T_{\pi }\left(G\right)$.

**Module Two: Online Inference.** The policy evaluation network $Q_{\pi_{g}}$, acquired through offline training, together with the state space S and action space A, collectively form the domain-theoretic representation $T_{\pi }\left(G\right)$. In accordance with Algorithm 2, during the online inference process, as the observation sequence O incrementally updates, we perform inference on all targets within the potential goal set G utilizing the domain-theoretic representation. Subsequently, we update the goal $g^{*}$ that provides the best fit to the observation sequence based on the distance evaluation method outlined in Equation (5).

This approach, employing an evaluation-based method with the aid of the assessment network, effectively mitigates the impact of observation sequence noise and missing data compared to the generative methods employed by planning-based goal recognition approaches.
**Algorithm 1** Offline Training for Domain Theory**Require:** S, A: State and action spaces in the continuous domain **Require:** map: A map used for navigation tasks in the continuous domain
 1:Initializing DRL (deep reinforcement learning) parameters γ 2:Initializing the reward function r(s,a) 3:Initializing training iterations *T* 4:**for** *t* = 1 to *T* **do** 5:   Randomly generating goal *g* in the map 6:   ∀a∀s≠g,r(s,a)←0 7:   ∀a,s=g,r(g,a)←C 8:   $Q_{\pi_{g}}\leftarrow DRL\left(S,A,\gamma \right)$ 9:**end for**10:**return** $T_{\pi }\left(G\right)$

**Algorithm 2** Online Infer most likely Goal for the Observations**Require:** $T_{\pi }\left(G\right)$: State S and action A spaces in the continuous domain, and policy evaluation networks $Q_{\pi_{g}}$ **Require:** G: a set of candidate goals **Require:** O: an observation sequence $O=\langle s_{0},a_{0},s_{1},a_{1},\ldots \rangle$
 1:Initializing minimum distance $\delta^{*}$ 2:**while** Observation sequence O update **do** 3:   **for** ∀g∈G **do** 4:     $\delta \leftarrow \mathrm{Distance}\left(Q_{\pi_{g}},O\right)$ 5:     **if** $\delta \leq \delta^{*}$ **then** 6:        $g^{*}\leftarrow g$ and $\delta^{*}\leftarrow \delta$ 7:     **end if** 8:   **end for** 9:**end while**10:**return** $g^{*}$


## 4. Goal Recognition as TD3

This section provides a comprehensive illustration of an online goal recognition algorithm in a continuous domain, leveraging the Twin Delayed Deep Deterministic Policy Gradient (TD3) algorithm, which is a deep reinforcement learning approach. We will systematically introduce the key principles of the TD3 algorithm, the essential parameter configurations for training the TD3 reinforcement learning algorithm, the rationale behind the reward function design, the convergence analysis of the TD3 algorithm training, and the measurement methodology for evaluating the policy evaluation network $Q_{\pi_{g}}$ against the observation sequence O.

### 4.1. Basic Principles of the TD3 Algorithm

The TD3 algorithm is an actor–critic reinforcement learning algorithm that extends the deep Q-network with a policy network, allowing direct output of action values. It represents an improvement over the Deep Deterministic Policy Gradient (DDPG) algorithm and is specifically designed for training environments with continuous action spaces.

**Figure 4:**
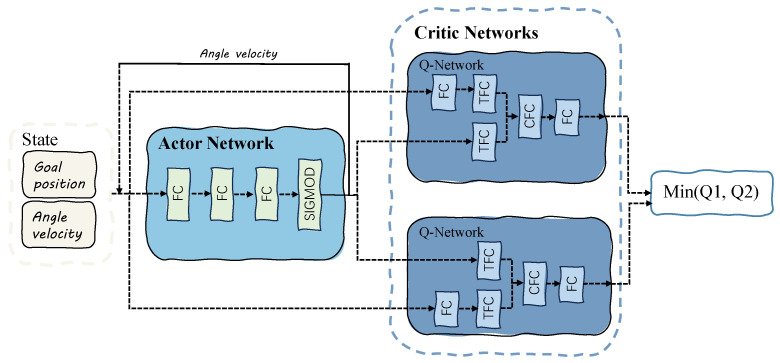
Network structure diagram of the TD3 algorithm.

Figure 4 illustrates the key characteristic of TD3, which lies in the utilization of twin critic networks. The algorithm simultaneously learns two Q-functions, $Q_{\phi_{1}}$ and $Q_{\phi_{2}}$, by minimizing the mean squared error. Both Q-functions share a single target, and the Q-target is selected as the smaller value between the outputs of the two Q-functions, as described below:

(6) $$y\left(r,s^{\prime },d\right)=r+\gamma \left(1-d\right)\min_{i=1,2}Q_{\phi_{i,\;\mathrm{targ}\;}}\left(s^{\prime },a_{\mathrm{TD}3}\left(s^{\prime }\right)\right)$$

Additionally, TD3 incorporates the concept of smoothing to enhance its performance. This is achieved by introducing noise to the target actions, which helps in smoothing the Q-values with respect to changes in actions. By doing so, TD3 makes it more difficult for the policy to exploit errors in the Q-function. The fundamental principle behind target policy smoothing can be summarized as follows:

(7) $$a_{\mathrm{TD}3\;}\left(s^{\prime }\right)=\mathrm{clip}\left(\mu_{\theta ,\mathrm{targ}}\left(s^{\prime }\right)+\mathrm{clip}\left(\epsilon ,-c,c\right),a_{\mathrm{low}\;},a_{\mathrm{high}\;}\right)$$

where ϵ is essentially noise, sampled from a normal distribution, i.e., $\epsilon ∼N\left(0,\sigma \right)$. Target policy smoothing is a regularization technique.

### 4.2. Parameter Design of the TD3 Algorithm’s Basic Structure

In the training process of the TD3 reinforcement learning algorithm within the simulation environment, Figure 4 demonstrates the inputs used, namely the polar coordinates of the agent’s goal, angular velocity ω, and linear velocity *v*. These inputs are combined to form the input state *s* for the actor network of the TD3 algorithm. The actor network consists of two fully connected (FC) layers, with Rectified Linear Unit (ReLU) activation applied after each layer. The final layer is connected to the output layer, which generates two action parameters: $a_{1}$ representing linear velocity and $a_{2}$ representing angular velocity of the robot. The output layer employs a hyperbolic tangent (tanh) activation function to ensure the values are constrained within the range of (−1,1). Before applying the action to the environment, it is scaled by the maximum linear velocity $v_{max}$ and maximum angular velocity $\omega_{max}$ to ensure the agent moves in the forward direction.

(8) $$a=\left[v_{max}\left(\frac{a_{1}+1}{2}\right),\omega_{max}a_{2}\right]$$

The TD3 algorithm evaluates the Q-value, denoted as Q(s,a), for a given state–action pair using two critic networks. These critic networks share the same structure, but their parameter updates are delayed, allowing for divergence in parameter values. For each critic network, the state–action pair (s,a) is provided as input. The state *s* is passed through a fully connected layer followed by ReLU activation, resulting in the output $L_{s}$. Additionally, the action *a* is also fed into a fully connected layer, resulting in the transformation of the action denoted as $\tau_{1}$ and $\tau_{2}$ for the two critic networks, respectively. The outputs from the transformation layers ($\tau_{1}$ and $\tau_{2}$) are then combined as follows:

(9) $$L_{c}=L_{s}W_{\tau_{1}}+aW_{\tau_{2}}+b_{\tau_{2}}$$

In the network architecture described, $L_{c}$ represents the combined fully connected layer (CFC), while $W_{\tau_{1}}$ and $W_{\tau_{2}}$ refer to the weights of the $\tau_{2}$ and $\tau_{2}$ transformation layers, respectively. $b_{\tau_{2}}$ represents the bias of the $\tau_{2}$ layer. After combining the outputs of the transformation layers, a Rectified Linear Unit (ReLU) activation function is applied to the combined layer. This activated layer is then connected to the output layer, which includes a parameter representing the Q-value. To mitigate the issue of overestimating the value of state–action pairs, the minimum Q-value from the two critic networks is selected as the final output of the critic. The complete network architecture, including the actor network and the twin critic networks, is visually presented in Figure 4.

The design of the reward function primarily encompasses three distinct scenarios: if the current number of time steps *t* to the goal’s distance falls below the threshold η, a positive target reward $r_{goal}$ is applied. In the event of a collision being detected, a negative collision reward $r_{coll}$ is applied. If neither of these conditions exist, a reward is immediately assigned based on the current linear velocity *v* and angular velocity ω.

(10) $$r\left(s_{t},a_{t}\right)=\left\{\begin{array}{cc} r_{goal} & \;\mathrm{if}\;t<\eta \\ r_{coll} & \;\mathrm{if}\;\mathrm{collision}\;\\ v-|\omega | & \;\mathrm{otherwise}\; \end{array}\right.$$

The policy network trained by the TD3 algorithm serves as the required policy evaluation network, denoted as $Q_{\pi_{g}}$, for the target recognition process. We employ measures, as proposed in the paper [11], to assess the distance between $Q_{\pi_{g}}$ and an observation sequence O. MaxUtil represents the cumulative utilities gathered from the observed trajectory.

(11) $$MaxUtil\left(Q_{\pi_{g}},O\right)=\sum_{i\in \left|O\right|}^{}Q_{\pi_{g}}\left(s_{i},a_{i}\right)$$

## 5. Experiment Evaluation

### 5.1. Offline Training of the TD3 Algorithm in ROS

**Figure 5:**
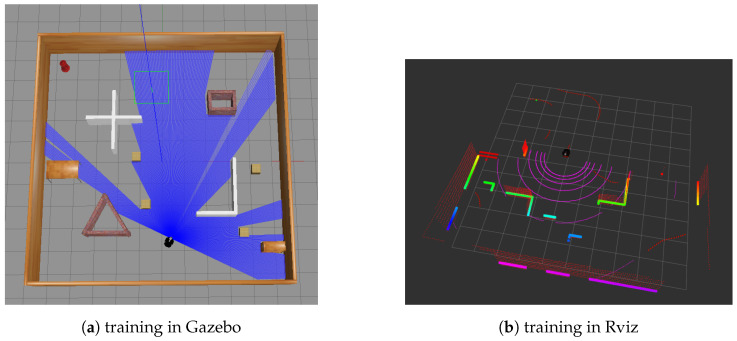
Navigation environment in ROS.

**Figure 6:**
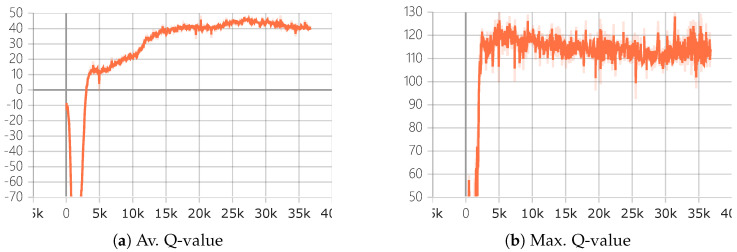
Q-value convergence curve. The x-axis represents the number of training iterations, measured in units of rounds, while the y-axis represents the reward Q-value.

In order to implement the goal recognition algorithm GR_DRL in an online navigation environment in a continuous domain, we conducted experiments on the ROS-based Gazebo platform. Initially, as depicted in Figure 5, training was carried out following the network architecture introduced in Chapter 4. Based on the training parameters we configured, we initiated training for a certain number of epochs. As illustrated in Figure 6, we provide the average and maximum values of Q-values, as well as the reward values. It can be observed that the training has converged, and the policy network obtained from this training serves as the required policy evaluation network, denoted as $Q_{\pi_{g}}$, for the goal recognition process.

### 5.2. Testing in a Continuous Domain

**Figure 7:**
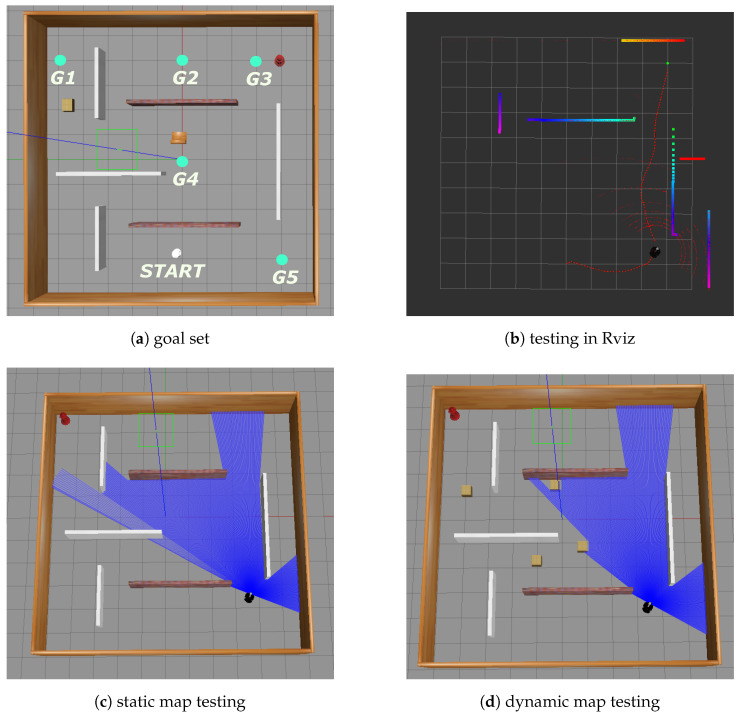
Testing in a continuous domain.

Based on the policy evaluation network $Q_{\pi_{g}}$ obtained through training, we acquired the domain-theoretic representation $T_{\pi }\left(G\right)$ for navigation tasks in a continuous domain. Subsequently, in the testing map shown in Figure 7a, we established a goal set G containing 5 potential goals $\left(G_{1},G_{2},G_{3},G_{4},G_{5}\right)$. We conducted tests to assess the recognition accuracy and online recognition speed of the deep reinforcement learning-based goal recognition algorithm in an online continuous environment, considering environmental dynamics, partially observable observation sequences, and noise interference in the observation sequences.

To evaluate recognition accuracy, we utilized common machine learning performance metrics, including accuracy, precision, recall, and F1score. These metrics are computed based on the combination of true positive (*TP*), true negative (*TN*), false positive (*FP*), and false negative (*FN*) instances with respect to the actual class labels and the corresponding model predictions, as described by the evaluation formulas.

Accuracy is defined as the ratio of correctly classified samples to the total number of samples. The corresponding formula is:

(12) $$ACC=\frac{TP+TN}{TP+TN+FP+FN}$$

Precision is defined as the ratio of true positives to the total number of predicted positive samples. The corresponding formula is:

(13) $$Pre=\frac{TP}{TP+FP}$$

Recall is defined as the ratio of true positives to the total number of actual positive samples. The corresponding formula is:

(14) $$Rec=\frac{TP}{TP+FN}$$

F1−score is derived from the harmonic mean of precision and recall. The corresponding formula is:

(15) $$F1=\frac{2Pre\times Rec}{Pre+Rec}$$

#### 5.2.1. Testing under Partial Observability

This study examines the impact of communication constraints on observation sequence missingness during online goal recognition. Specifically, we investigate two distinct sources of partial observability: varying sampling rates and sensor failures. The key differentiation lies in the uniformity of partial observability caused by varying sampling rates, while temporary sensor failures result in continuous periods of missing data. To comprehensively evaluate this phenomenon, we conducted experiments considering five different levels of observability (5%, 10%, 30%, 50%, and full observability). Additionally, random obstacles were introduced to assess the algorithm’s robustness in both dynamic and static environmental conditions. Each experiment was repeated 100 times, and the resulting data are presented in Table 1. These findings contribute valuable insights into the algorithm’s performance under communication constraints and shed light on the varying levels of observability.

According to the observations, the recognition algorithm demonstrates remarkable accuracy, surpassing 90%, when confronted with both types of observation missingness (varying sampling rates and sensor failures) in static environments. Even in dynamic environments, where some challenges are present, the algorithm exhibits a commendable recognition accuracy of over 80%, showcasing its robustness in coping with the complexities of changing environmental conditions.

#### 5.2.2. Testing under Observation Sequence Noise

In the realm of online goal recognition tasks, communication frequently contends with diverse degrees of noise interference. Conventional forms of communication noise are typically categorized into three primary classes: **Gaussian white noise**, **Laplace noise**, and **Poisson noise**. Within the scope of this investigation, precision assessments were executed on observation sequences exhibiting distinct signal-to-noise ratios (SNRs). These assessments encompassed two distinct observability states: 50% and full observability. The evaluations took into account both dynamic and static environmental scenarios. The SNR calculation formula is articulated as follows:

(16) $$db=10\log\left(\frac{s}{n}\right)$$

In this context, *s* represents the signal, and *n* represents the noise. The signal-to-noise ratio (SNR) is measured in decibels (dB) and is computed as follows:

(17) $$db=10\log\left(\frac{P_{s}}{P_{n}}\right)$$

where $P_{s}$ and $P_{n}$ respectively denote the effective power of the signal and noise. In general, a higher signal-to-noise ratio (SNR) corresponds to higher audio playback quality, indicating that the noise mixed with the signal is lower. Conversely, a lower SNR results in lower audio quality. To ensure the quality of sound propagation, the SNR should typically not fall below 70 dB. However, to simulate robust adversarial scenarios, in the experiments of this section, extreme cases of 80 dB, 50 dB, and 10 dB were selected for analysis. The experimental results are presented in Table 2.

It can be observed that across the tests involving three different types of noise, in both dynamic and static environmental conditions, the recognition accuracy consistently exceeds 60%. Furthermore, under various levels of signal-to-noise ratio (SNR) interference, the overall accuracy exhibits relatively minor fluctuations. In the extreme scenario with 50% observability and an SNR of 10 dB, Gaussian noise, Laplace noise, and Poisson noise all achieve recognition accuracies of 88%, 78%, and 61% or higher, respectively.

#### 5.2.3. Online Recognition Speed Testing

The recognition speed in online goal recognition, especially in the presence of communication interference, is an essential metric to consider. We conducted tests on the online recognition speed under two different environmental conditions (static and dynamic) for five levels of partially observable scenarios (5%, 10%, 30%, 50%, and full observability), where observability is affected by varying sampling rates. In this study, the successful recognition time was determined as the time taken to correctly identify the target within three consecutive time steps. The experimental results are presented in Table 3.

Additionally, we evaluated the online recognition speed under the influence of Gaussian noise interference at three different signal-to-noise ratios (10 dB, 50 dB, 80 dB). These tests were conducted for two observability levels (50% and full observability). The experimental results are shown in Table 4.

According to Table 3, it is evident that the impact of five different levels of missing observation sequences on online recognition speed remains within one second. Furthermore, the influence of environmental conditions, both static and dynamic, on recognition speed is also quite limited.

Moreover, from Table 4, it can be observed that the impact of Gaussian noise under different observability levels (50% and full observability) on recognition speed can be considered negligible. Similarly, the influence of noise at different signal-to-noise ratios (10 dB, 50 dB, 80 dB) on recognition speed is kept within 1 second. Even in scenarios with partial observability and Gaussian noise interference, the recognition accuracy still maintains a level exceeding 90%.

## 6. Discussion and Conclusions

In this paper, we presented a continuous-domain online real-time goal recognition algorithm based on the TD3 deep reinforcement learning algorithm. The algorithm was validated through experiments conducted on the ROS-based Gazebo simulation platform. We examined the robustness of the goal recognition algorithm under various communication constraints, including partial observability and observation noise, and also evaluated the online recognition speed.

Our approach extends traditional reinforcement learning-based goal recognition algorithms to the continuous domain, addressing the challenge of representing all action–state pairs when the action and state spaces are infinite. By employing deep reinforcement learning, we learned the agent’s policy evaluation network, denoted as $Q_{\pi_{g}}$, which, together with the continuous action space A and state space S, forms a domain-theoretic representation $T_{\pi }\left(G\right)$ based on neural networks. This representation successfully captures the Q-values for all action–state pairs in continuous spaces. Subsequently, we employed the MaxUtil method proposed in Goal Recognition As Q-Learning [11] to infer the most likely goal $g^{*}$ based on the maximum Q-value.

Differing from generative approaches proposed by planning-based goal recognition methods, our reinforcement learning-based algorithm adopts an evaluative approach, implicitly mitigating the impact of observation noise and sequence missingness on recognition accuracy. Our experiments also demonstrated the algorithm’s excellent resistance to disturbances and robustness in real-world, communication-constrained scenarios. Furthermore, our offline learning and online inference approach significantly enhances the speed of online recognition, compared to planning-based recognition methods that require re-invoking planners for every new observation sequence.

In summary, our work represents a significant advancement in the field of goal recognition. While this paper primarily focuses on goal recognition in continuous navigation environments, the proposed approach can be applied to a wide range of tasks in continuous domains in real-world environments. Examples include goal inference for robotic arm grasping tasks and tactical maneuvers for fighter aircraft. As long as we employ deep reinforcement learning to train a policy network model tailored for the respective task during offline learning and utilize the framework introduced in this paper to construct domain-theoretic representations $T_{\pi }\left(G\right)$ based on neural networks, we can successfully address various online goal recognition tasks. The paper [4,5] demonstrates the extensive prospects of goal recognition in the field of autonomous driving. The approach presented in this paper exhibits excellent performance on a continuous domain platform based on Gazebo. Future research could consider testing and applying it on autonomous driving simulation platforms like Carla.

## Figures and Tables

**Table 1:** Impact of partial observability (5%, 10%, 30%, 50%, and full observability).

Partial OBS		acc		prec		rec		f-s	
	Type	Dynamic	Statics	Dynamic	Statics	Dynamic	Statics	Dynamic	Statics
5%	sampling rates	0.90	**0.94**	0.74	**0.86**	0.74	**0.86**	0.74	**0.86**
10%	sampling rates	0.92	**0.96**	0.79	**0.89**	0.79	**0.89**	0.79	**0.89**
30%	sampling rates	0.92	**0.96**	0.79	**0.89**	0.79	**0.89**	0.79	**0.89**
50%	sampling rates	0.92	**0.96**	0.79	**0.89**	0.79	**0.89**	0.79	**0.89**
100%	sampling rates	0.94	**0.96**	0.84	**0.89**	0.84	**0.89**	0.84	**0.89**
**avg**		**0.92**	**0.95**	**0.79**	**0.88**	**0.79**	**0.88**	**0.79**	**0.88**
5%	sensor failures	0.82	**0.83**	0.56	**0.58**	0.58	**0.60**	0.57	**0.59**
10%	sensor failures	0.86	**0.89**	0.66	**0.72**	0.66	**0.72**	0.66	**0.72**
30%	sensor failures	0.87	**0.90**	0.68	**0.76**	0.68	**0.76**	0.68	**0.76**
50%	sensor failures	0.87	**0.90**	0.68	**0.76**	0.68	**0.76**	0.68	**0.76**
100%	sensor failures	0.92	**0.96**	0.79	**0.89**	0.79	**0.89**	0.79	**0.89**
**avg**		0.87	0.90	0.67	0.74	0.68	0.75	0.68	0.74

**Table 2:** Impact of noise observability (Gaussian noise, Laplace noise, Poisson noise).

Noise OBS			acc		prec		rec		f-s	
	Type	SNR	Dynamic	Statics	Dynamic	Statics	Dynamic	Statics	Dynamic	Statics
Gaussian noise	full obs	10 dB	0.92	**0.96**	0.81	**0.90**	0.81	**0.90**	0.81	**0.90**
		50 dB	0.93	**0.97**	0.82	**0.93**	0.82	**0.93**	0.82	**0.93**
		80 dB	0.93	**0.97**	0.82	**0.93**	0.82	**0.93**	0.82	**0.93**
Gaussian noise	50% obs	10 dB	0.88	**0.92**	0.69	**0.80**	0.69	**0.80**	0.69	**0.80**
		50 dB	0.92	**0.97**	0.81	**0.92**	0.81	**0.92**	0.81	**0.92**
		80 dB	0.92	**0.97**	0.81	**0.93**	0.81	**0.93**	0.81	**0.93**
Laplace noise	full obs	10 dB	0.83	**0.92**	0.57	**0.80**	0.57	**0.80**	0.57	**0.80**
		50 dB	0.84	**0.94**	0.60	**0.84**	0.60	**0.84**	0.60	**0.84**
		80 dB	0.86	**0.96**	0.64	**0.90**	0.64	**0.90**	0.64	**0.90**
Laplace noise	50% obs	10 dB	0.78	**0.88**	0.46	**0.71**	0.46	**0.71**	0.46	**0.71**
		50 dB	0.82	**0.91**	0.54	**0.77**	0.54	**0.77**	0.54	**0.77**
		80 dB	0.84	**0.94**	0.59	**0.84**	0.59	**0.84**	0.59	**0.84**
Poisson noise	full obs	10 dB	0.86	**0.95**	0.64	**0.87**	0.64	**0.87**	0.64	**0.87**
		50 dB	0.93	**0.96**	0.82	**0.90**	0.82	**0.90**	0.82	**0.90**
		80 dB	0.94	**0.97**	0.84	**0.93**	0.84	**0.93**	0.84	**0.93**
Poisson noise	50% obs	10 dB	0.61	**0.60**	0.02	**0.00**	0.02	**0.00**	0.02	**0.00**
		50 dB	0.92	**0.60**	0.81	**0.00**	0.81	**0.00**	0.81	**0.00**
		80 dB	0.93	**0.95**	0.82	**0.87**	0.82	**0.87**	0.82	**0.87**

**Table 3:** Online goal recognition speed under partial observability.

Online Recognition	acc		Time(s)	
	Dynamic	Statics	Dynamic	Statics
**5%**	0.90	**0.94**	10.68	**10.17**
**10%**	0.92	**0.96**	10.55	**9.90**
**30%**	0.92	**0.96**	10.54	**9.73**
**50%**	0.92	**0.96**	10.53	**9.67**
**100%**	0.94	**0.96**	10.51	**9.66**
**avg**	0.92	**0.95**	10.56	**9.83**

**Table 4:** Online goal recognition speed under Gaussian noise.

Gaussian Noise		acc		Time(s)	
	Partial OBS	Dynamic	Statics	Dynamic	Statics
**10 db**	**full obs**	0.92	**0.96**	11.00	**10.30**
**50 db**		0.93	**0.97**	10.86	**10.12**
**80 db**		0.93	**0.97**	10.63	**10.00**
**avg**		0.93	**0.97**	10.83	**10.14**
**10 db**	**50% obs**	0.92	**0.96**	11.18	**10.83**
**50 db**		0.93	**0.97**	11.17	**10.72**
**80 db**		0.93	**0.97**	11.07	**10.72**
**avg**		0.93	**0.97**	11.14	**10.76**

## Data Availability

Data supporting the reported results can be found at https://github.com/pucrs-automated-planning/graql_aaai, Deep reinforcement learning algorithms can be found at https://github.com/datawhalechina/easy-rl and the ROS experimental environment can be found at https://www.theconstructsim.com/robotigniteacademy_learnros/ros-courses-library/using-openai-with-ros-online-course/, all accessed on 29 August 2023.

## Abbreviations

The following abbreviations are used in this manuscript:

GR_DRL	Goal Recognition as Deep Reinforcement Learning
GR	Goal Recognition
PRAP	Plan Recognition as Planning
PDDL	Planning Domain Definition Language
XGBoost	eXtreme Gradient Boosting
LSTM	Long Short-Term Memory networks
TD3	Twin Delayed Deep Deterministic Policy Gradient
DDPG	Deep Deterministic Policy Gradient
FC	Fully Connected
ReLU	Rectified Linear Unit
CFC	Combined Fully Connected Layer
SNR	Signal-to-Noise Ratio
